# Selection of reference genes for diurnal and developmental time-course real-time PCR expression analyses in lettuce

**DOI:** 10.1186/s13007-016-0121-y

**Published:** 2016-03-22

**Authors:** Tiziana Sgamma, Judith Pape, Andrea Massiah, Stephen Jackson

**Affiliations:** School of Life Sciences, University of Warwick, Gibbet Hill Road, Coventry, West Midlands CV4 7AL UK; Faculty of Health and Life Sciences, De Montfort University, The Gateway, Leicester, LE1 9BH UK

**Keywords:** Reference gene, Normalisation, qRT-PCR, Lettuce, Flowering time, FT

## Abstract

**Background:**

Real-time quantitative polymerase chain reaction (RT-qPCR) analysis is a low cost and sensitive technique that is widely used to measure levels of gene expression. Selecting and validating appropriate reference genes for normalising target gene expression should be the first step in any expression study to avoid inaccurate results.

**Results:**

In this study, ten candidate genes were tested for their suitability for use as reference genes in diurnal and developmental timecourse experiments in lettuce. The candidate reference genes were then used to normalise the expression pattern of the *FLOWERING LOCUS T* (*FT*) gene, one of key genes involved in the flowering time pathway whose expression is known to vary throughout the day and at different stages of development. Three reference genes, *LsPP2A*-*1* (*PROTEIN PHOSPHATASE 2A*-*1*), *LsPP2AA3* (*PROTEIN PHOSPHATASE 2A REGULATORY SUBUNIT A3*) and *LsTIP41* (*TAP42*-*INTERACTING PROTEIN OF 41* *kDa*), were the most stably expressed candidate reference genes throughout both the diurnal and developmental timecourse experiments. In the developmental experiment using just *LsPP2A*-*1* and *LsTIP41* as reference genes would be sufficient for accurate normalisation, whilst in the diurnal experiment all three reference genes, *LsPP2A*-*1*, *LsPP2AA3* and *LsTIP41*, would be necessary. The *FT* expression pattern obtained demonstrates that the use of multiple and robust reference genes for RT-qPCR expression analyses results in a more accurate and reliable expression profile.

**Conclusions:**

Reference genes suitable for use in diurnal and developmental timecourse experiments in lettuce were identified and used to produce a more accurate and reliable analysis of *lsFT* expression levels than previously obtained in such timecourse experiments.

**Electronic supplementary material:**

The online version of this article (doi:10.1186/s13007-016-0121-y) contains supplementary material, which is available to authorized users.

## Background

One of the most common techniques for studying gene expression is real-time quantitative polymerase chain reaction (RT-qPCR) analysis because it is extremely sensitive, specific and cost-effective. However there are often methodological errors in RT-qPCR application and, as a consequence, in the interpretation of the results [1]. One of the most common errors is the choice of inappropriate reference genes used to normalise the expression of target genes [1–3]. Ideally reference genes would be expressed at a constant level and would thus be representative of the cDNA concentration in each sample, but their expression is often affected by experimental treatments or can vary in different tissues, or over the time-course chosen for the experiment [4]. In literature there are several genes that are commonly used as reference genes but results often show that each experimental design and each organism requires its own reference genes to be selected and used [2, 3, 5]. Several approaches can be used for a systematic validation of reference genes such as NormFinder, Best-keeper and geNorm [6–8]. These software-based approaches rank a plethora of candidate reference genes measuring their relative stability by comparing the expression of each gene in each sample in relation to the entire set of reference genes. The best approach is to follow the Minimum Information for Publication of Quantitative Real-Time PCR Experiments (MIQE) guidelines and to use multiple reference control genes, as the use of single reference gene is now considered inappropriate [9].

Recently, a study investigated 17 selected candidate genes and microRNAs (miRNAs) for their suitability to be used as reliable and stable reference genes in RTqPCR analysis of gene expression differences in abiotic stress experiments [10]. Some of these were genes such as *TAP42*-*INTERACTING PROTEIN OF 41* *kDa* (*TIP41*), *ADENOSINE PHOSPHORIBOSYL TRANSFERASE 1* (*APT1*) and miRNA genes and were selected on the basis that they had been used as reference genes in previously published work [2, 11–13]. The results of the experiments using abiotic stresses such as drought, salinity, UV-C irradiation, heavy metal stress and abscisic acid treatments, showed differences in stability between the reference genes [10]. Therefore in any RTqPCR experiment the identification of a good set of reference genes whose expression remains constant throughout the experimental conditions under study is extremely important in order to be able to obtain reliable results.

Lettuce (*Lactuca sativa*) is a leafy vegetable cultivated throughout the course of the year, it is harvested when plants reach maturity but before they bolt and flower [14]. In order to avoid harvest loss and wastage it is therefore important to be able to predict and anticipate when the crop will start bolting. Understanding changes in gene expression profiles, in particular those of flowering time genes, throughout development will help to develop predictable cropping.

In this study we wanted to identify suitable reference genes that would have stable expression profiles over diurnal and developmental timecourse experiments in order to analyse the expression of flowering time genes over these timecourses. Ten reference genes were selected based on genes that had been identified in previous experiments in other species and the stability of their expression profiles over developmental and diurnal timecourses was tested. To demonstrate the reliability and suitability of the reference genes identified, the expression pattern of the *FLOWERING LOCUS T* (*FT*) gene, one of key genes involved in the flowering time pathway, was analysed in lettuce leaves sampled over the diurnal and developmental timecourse experiments using the selected reference genes for normalisation.

## Results

### PCR amplification efficiency and expression profiling of candidate reference genes

A total of 10 genes were selected as good potential candidates for reference genes for normalisation of gene expression over diurnal and developmental timecourse experiments based on previous reports of their use as reference genes in other species (Table 1), and on the availability of lettuce homologues in the expressed sequence tag (EST) Compositae Genome Project Database (CGPDB 2013; http://cgpdb.ucdavis.edu/).

**Table 1:** Candidate reference genes selected for testing

Gene symbol	Definition	*Arabidopsis thaliana* homolog locus tag	References	Lettuce Genbank ID
UBQ1	Putative ubiquitin extension protein	AT3G52590.1	[41]	NM_129273.4
UBQ7	Ubiquitin-like protein RUB2	AT2G35635	[36]	NM_129118
ACT2	Actin 2	At3g18780	[5]	AK317453.1
ACT12	Actin protein coding 12	At3G46520	[37]	BT005073.1
TUA-3	Tubulin alpha-3	At5g19770	[38]	BT000718.1
GAPDH	Glyceraldehyde 3-phosphate dehydrogenase, cytosolic	At1g13440	[5, 37, 40]	AK317337.1
UBC9	Ubiquitin conjugating enzyme 9	At4g34270	[39]	AF325019.2
TIP41	TIP41-like protein	AT4G34270	[5, 11, 39, 42]	NM_119592.4
PP2AA3	Protein phosphatase 2A regulatory subunit A3	At1g13320	[5, 39]	BT002601.1
PP2A-1	Protein phosphatase 2A-1	At1g59830	[5]	AY096543.1

Primers were designed to the homologous lettuce genes. The optimum primer concentration and temperature were determined for each primer pair (Additional file 1: Table S1). The specificity of each primer set was validated by obtaining just a single melting curve peak for each candidate gene after 40 Real-time quantitative PCR cycles with no signal in the negative samples (Additional file 2: Figure S1). PCR products were sequenced to confirm the identity of the amplified target sequence. The correlation coefficient (R^2^) for quality assays is usually set as >0.980 and it represents how well the tested samples fit the regression line generated by the standard curve [15]. All primer pairs, except LsUBQ7F/R and LsPP2A-1F/R, showed an R^2^ higher than 0.980 ranging between 0.982 and 0.996 (Table 2). The amplification efficiency (E) indicates the amplification rate of a template during the reaction. The theoretical optimum value is 100 % indicating that the template duplicates in an exponential way and the acceptable range is usually set between 90 and 110 %. [9]. For the 10 reference genes tested the efficiency varied from 90.3 and 105.9 % (Table 2). Therefore, all the primer pairs were deemed sufficiently well designed to use in RT-qPCR experiments.

**Table 2:** Candidate reference gene performance at optimum concentration

Primers	E (%)	R^2^	Amplification factor
LsUBQ1F/R	94.9	0.992	1.95
LsUBQ7F/R	97.3	0.945	1.97
LsACT2F/R	91.3	0.993	1.91
LsACT12F/R	94.0	0.996	1.94
LsTUA-3F/R	90.3	0.994	1.90
LsGAPDHF/R	91.9	0.991	1.92
LsUBC9F/R	98.0	0.986	1.98
LsTIP41F/R	91.6	0.996	1.92
LsPP2AA3F/R	96.8	0.982	1.97
LsPP2A‐1F/R	105.9	0.920	2.06

**Fig. 1:**
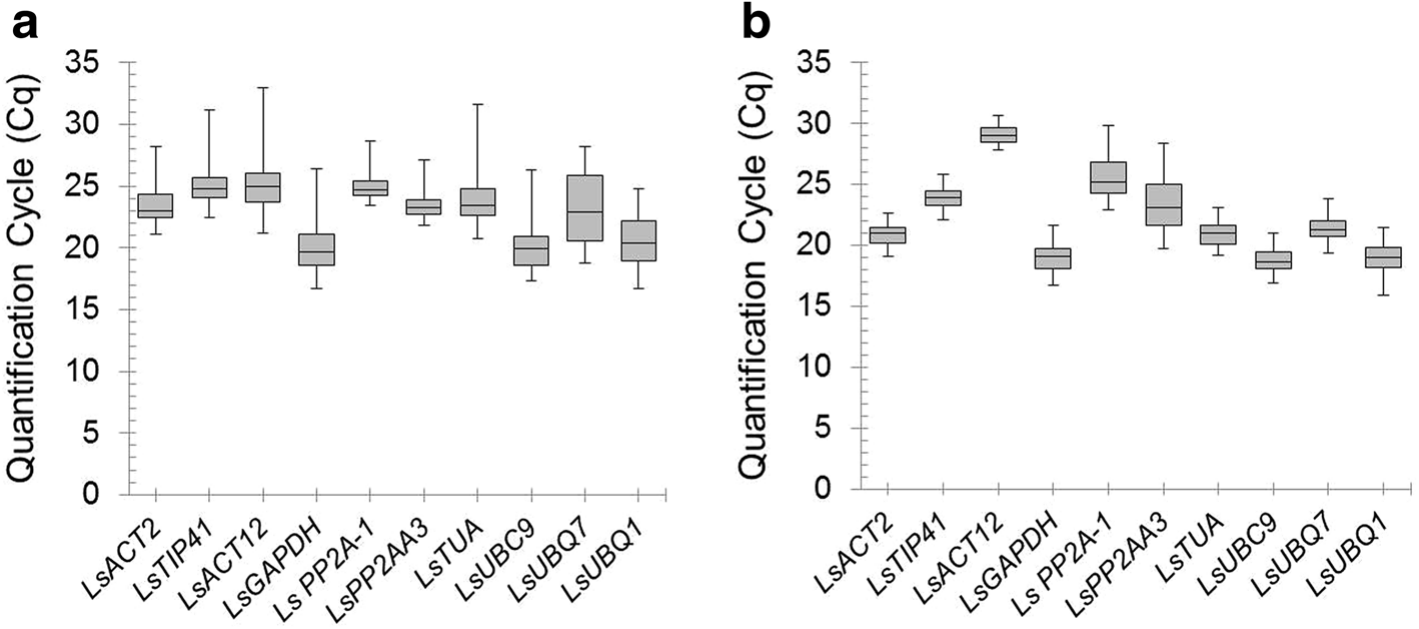
Expression levels of candidate reference genes over **a** diurnal and **b** developmental timecourse experiments. The values are given as real-time PCR quantification cycle (Cq) values for individual reference genes. *Boxes* indicate the interquartile range and the line across the *box* depicts the median. Whiskers represent 95 % confidence intervals

The quantification cycle (Cq) value determined the relative expression levels of the candidate reference genes. The Cq value was used to compare the expression levels of the 10 candidate reference genes in the diurnal and developmental experimental samples. The expression stability of each candidate reference gene is presented in Fig. 1. In the diurnal experiment the Cq value ranged between 16.7 and 32.9, while in the developmental experiment ranged between 15.9 and 30.6. In both diurnal and developmental timecourses *UBC1, GAPDH* and *UBC9* had the highest level of expression, i.e. showed the lowest Cq values with a mean of 20.45, 20.01, 20.03 and 18.99, 18.92, 18.79 in the diurnal and developmental timecourse experiments respectively. *LsACT12* was the lowest expressed of the reference genes tested in both experiments showing a Cq mean of 25.07 and 29.07 for the diurnal and developmental timecourse experiments respectively. Over the diurnal timecourse, *LsPP2A*-*1* showed the lowest level of variation in gene expression (coefficient of variation, CV, 4.6 %), while the *LsUBQ7* was the most variable (CV, 12.3 %). Over the developmental timecourse *LsACT12* was the least variable across the Saladin samples (CV, 2.7 %), while *LsPP2AA3* was the most variable (CV, 9.6 %).

### Stability of candidate reference genes

**Fig. 2:**
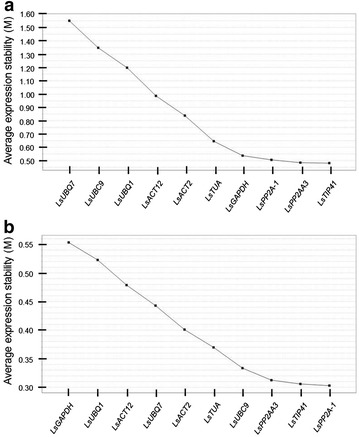
Average expression stability value (M) calculated by geNORM. **a** M of different candidate reference genes over diurnal timecourse samples. **b** M of different candidate reference genes over developmental timecourse samples

The stability of the 10 candidate reference genes was evaluated using geNorm software to calculate the gene average expression stability value (M). GeNorm’s threshold for eliminating a gene as unstable was set as M ≥ 1.5 [7]. The expression stability of the candidate reference genes was analysed over both diurnal and developmental timecourse experiments (Fig. 2). The trend observed showed that the selected candidate reference genes were generally more stable over the developmental timecourse experiment than in the diurnal timecourse experiment. Nevertheless, all the candidate reference genes, with exception of *LsUBQ7* in the diurnal experiment, had an M value ≤1.5. Interestingly, although the ranking in the two experiments was different, the three most stable candidate reference genes were the same. In each experiment, *LsPP2A*-*1*, *LsPP2AA3* and *LsTIP41* had the lowest M value, indicating that these three genes were the most suitable reference genes to use for normalization in diurnal and developmental timecourse experiments in lettuce.

**Fig. 3:**
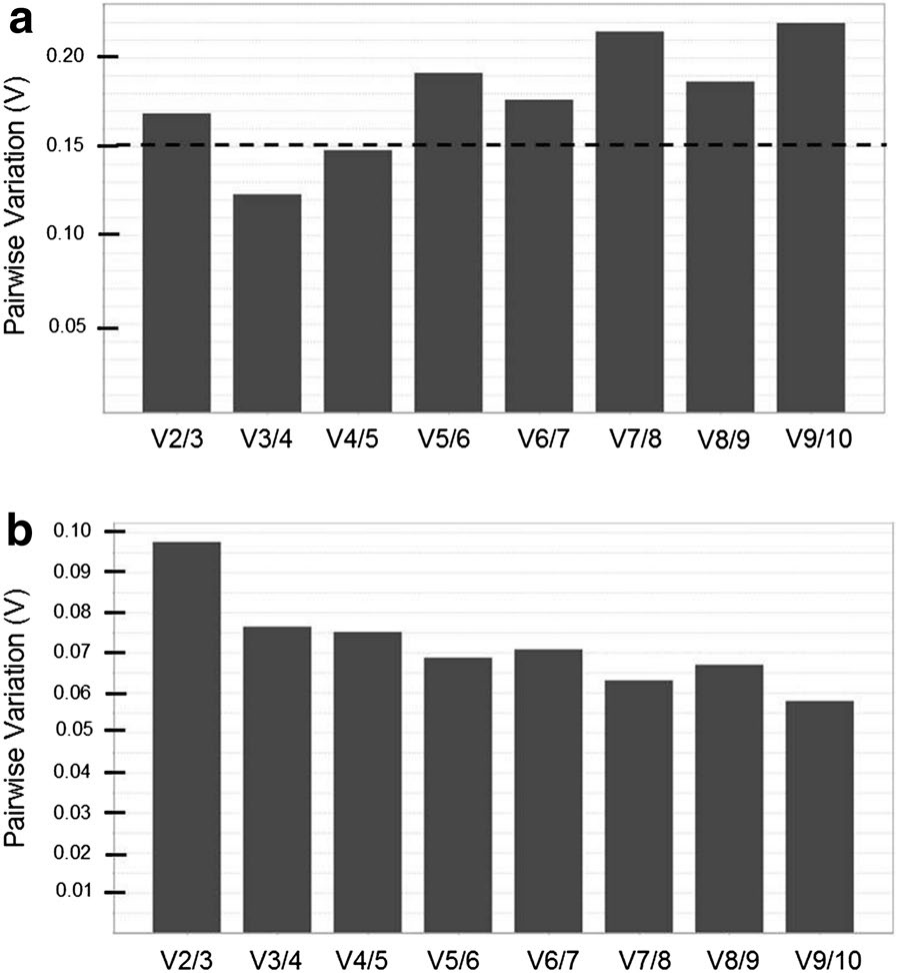
Determination of the optimal number of reference targets required for normalization. **a** Pairwise variation (V) of candidate reference genes in diurnal timecourse samples. **b** V of candidate reference genes in developmental timecourse samples. The pairwise variation (V_n_/V_n+1_) was analyzed between the normalization factors NF_n_ and NF_n+1_ by the geNorm software to determine the optimal number of reference genes required for qRT-PCR data normalization. The *dashed line* indicates the 0.15 threshold

GeNorm also provides information about the optimum number of reference genes to be used in the experiment according to the pairwise variation (V). A cut-off of 0.15 is usually applied [7]. In the diurnal timecourse experiment, the optimum number of references genes to use is 3 (geNorm V < 0.15) (Fig. 3a). In this experimental situation the optimal normalization factor would be calculated as the geometric mean of the expression of the reference genes *LsPP2A*-*1*, *LsPP2AA3* and *LsTIP41*. In the developmental timecourse experiment, it would be fine to use just two reference genes (geNorm V < 0.15) (Fig. 3b). In this case the reference genes *LsPP2A*-*1* and *LsTIP41* would be sufficient for accurate normalization.

### Validation of the reference genes

*FT* is one of the key genes involved in the flowering time pathway whose expression varies over a diurnal and developmental timecourse. We used the lettuce *FT* homologue (*LsFT*) to evaluate the reliability of the selected reference genes to normalise its expression in diurnal and developmental timecourse RT-qPCR analyses. *LsPP2A*-*1*, *LsPP2AA3* and *LsTIP41* were used as optimal reference genes in the diurnal time-course. *LsPP2A*-*1* and *LsTIP41* were used as optimal reference genes in the developmental time-course.

**Fig. 4:**
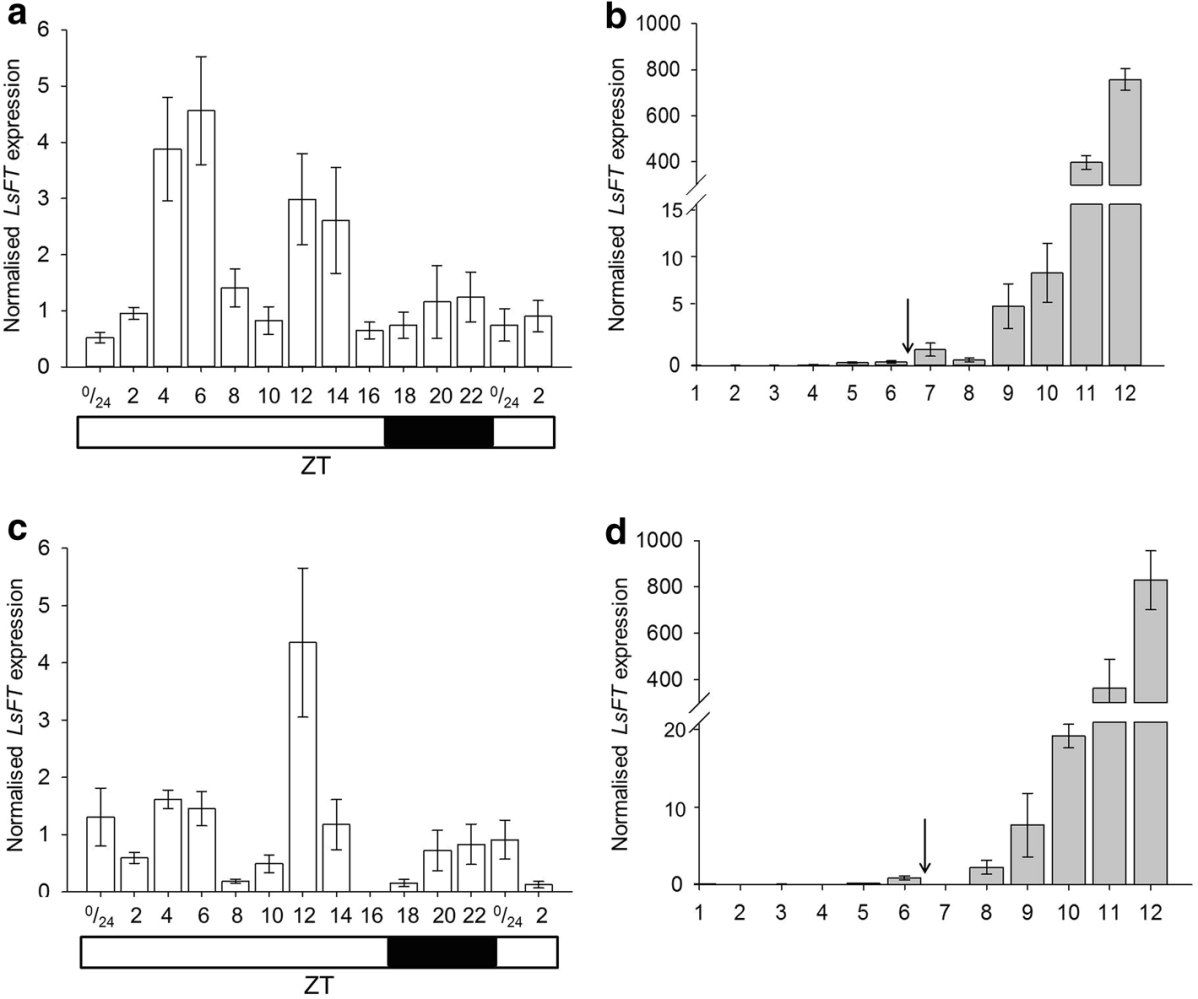
Normalised expression levels of *LsFT*. **a** *LsFT* expression levels normalised using the three most stable reference genes, *LsPP2A*-*1*, *LsPP2AA3* and *LsTIP41*, over the diurnal time-course. The *white bar* *below* indicates lights on, the *black bar* indicates darkness. **b** *LsFT* expression levels normalised against the two most stable reference genes, *LsPP2A*-*1* and *LsTIP41*, over the developmental time-course. Samples were collected once a week at 14 h after dawn. The *arrow* indicates when the plants bolted. **c** *LsFT* expression levels normalised against three of the least-stable reference genes, *LsUBQ1*, *LsUBQ7* and *LsACT12*, over the diurnal time-course. **d** *LsFT* expression levels normalised against three of the least-stable reference genes, *LsUBQ1*, *LsUBQ7* and *LsACT12*, over the developmental time-course

In the diurnal time-course normalised *LsFT* expression exhibited two peaks, one at around ZT 4–6 and another around ZT 12–14 (Fig. 4a). In the developmental timecourse experiment, *LsFT* starts to increase two weeks after bolting at sample 9 and a further substantial increase happened after this point reaching a very high level 28 days after bolting (Fig. 4b).

To illustrate the difference in the *LsFT* expression pattern that would be obtained if sub-optimal reference genes were used for normalisation, three commonly used (but the less stable) reference genes, *LsUBQ1*, *LsUBQ7* and *LsACT12* were used for a comparative analysis (Fig. 4c, d). In the diurnal time-course normalised *LsFT* expression exhibited a different pattern from Fig. 4a with only one peak, around ZT 12 (Fig. 4c). There was, however, little difference observed in the developmental timecourse experiment (Fig. 4b, d) which reflects the overall greater stability of all the candidate reference genes in this experiment compared to the diurnal experiment (Fig. 2a, b).

To validate the usefulness of *LsPP2A*-*1*, *LsPP2AA3* and *LsTIP41* as good reference genes for diurnal experiments, the expression level of another target gene, the lettuce *FLAVIN*-*BINDING, KELCH REPEAT, F*-*BOX* (*FKF1*) homologue (*LsFKF1*) [43], was studied in the diurnal time-course experiment. In the diurnal time-course normalised *LsFKF1* expression increased from ZT 8 reaching a maximum level at ZT 12, after this point the expression decreased dramatically reaching very low level just before the dark period (Additional file 3: Figure S2).

## Discussion

Quantifying gene transcripts using RT-qPCR has become a very widespread technique and there is a great need for a reliable method to analyse and interpret the results [1]. One of the main problems is selecting reliable reference genes to use for normalisation of the expression of the target gene [1–3].

Recently, the need to identify reliable reference genes was presented in a study by Borowski et al. [10] where 17 candidates were studied for their suitability as reference genes in abiotic stress experiments. Results showed that different abiotic treatments required different reference genes as the stability of the genes was different in different experimental situations. The identification of stable reference genes in different experimental contexts is therefore essential in order to obtain accurate and reliable gene expression data.

In the present study a geNorm approach was used to investigate 10 candidate genes for their potential to be used as reference genes in diurnal and developmental time-course expression experiments in lettuce. GeNorm M values showed that three reference genes, *LsPP2A*-*1*, *LsPP2AA3* and *LsTIP41*, were more constantly expressed throughout both the diurnal and developmental timecourse experiments. These reference genes were stable and constantly expressed in all the samples showing that they are not influenced by developmental stage (even the onset of flowering) or diurnal cycles in lettuce. The geNorm V values suggested that in the developmental experiment using just two reference genes *LsPP2A*-*1* and *LsTIP41* would be sufficient for accurate normalisation, whilst in the diurnal experiment three reference genes (*LsPP2A*-*1*, *LsPP2AA3* and *LsTIP41*) would be necessary.

Flowering is one of the major developmental changes that plants undergo during their development. It is tightly regulated and involves many changes in metabolic and developmental pathways [16–18]. Therefore finding reference genes that do not fluctuate during this process that can be used as reference genes to examine the expression of genes involved in the flowering pathway is very important. PP2AA3, is a catalytic subunit of serine/threonine protein phosphatase or PP2A-1, an enzyme that remove phosphatase groups from the given substrate [19, 20]. TIP-41 is a tonoplastic intrinsic protein, a type of membrane protein channel that allows the movement of water and amino acids from the tonoplastic interior to the cytoplasm [21]. The constant expression of these genes throughout development and during the day is probably due to the essential role they play in cells at all developmental stages. We can speculate that these reference genes could be good candidate reference genes for similar time course studies in other species, for example it is interesting to note that these three reference genes (*LsPP2A*-*1*, *LsPP2AA3* and *LsTIP41*) were also identified as three of the most stable genes in Affymetrix GeneChip developmental timecourse data [5], but the stability of these candidate reference genes would still need to be checked for particular species and the specific experimental conditions used.

To demonstrate that these genes are good reference genes for RT-qPCR diurnal and developmental timecourse experiments in lettuce, the expression profile of *LsFT* was studied in these timecourse experiments. *FT* has been isolated and studied in several plants as well as in lettuce [22–27], and it has been shown to be repressed during the juvenile phase and promoted during the adult vegetative phase of development [28, 29]. In *Arabidopsi*s, *FT* expression is promoted principally by CONSTANS at the end of a long day but other diurnal patterns have been observed in other plants [30–34]. In lettuce *LsFT* expression has been studied during development in sequentially growing leaves showing an increase in expression. *FT* diurnal expression was also studied and it showed a peak in expression at the beginning and at the end of the day with a trough in expression at ZT = 6 [27]. These RTqPCR experiments however used only one reference gene, beta-tubulin (*BTUB1*), to normalise *FT* expression. Our results using the averaged value of three reference genes revealed a different *FT* diurnal expression pattern which peaks twice during the day at ZT 4–6 and at ZT 12–14 (Fig. 4a). This expression pattern differs to the diurnal pattern of *LsFT* previously observed by Fukuda et al. [27] and thus demonstrates the value of selecting multiple robust reference genes for RT-qPCR expression analyses.

To demonstrate that a poor selection of reference genes can affect the expression pattern obtained, three of the less stable reference genes from our analysis, *LsUBQ1*, *LsUBQ7* and *LsACT12* were used in a comparative analysis of the expression profile of *LsFT* in the same timecourse experiments (actin and ubiquitin being reference genes used most routinely in RT-qPCR studies [10]). This showed that using reference genes with low stability and high M values (such as *LsUBQ1*, *LsUBQ7* and *LsACT12* in the diurnal experiment) will give a very different outcome, however if they have low M values as in the developmental experiment where they were more stable then the result is not greatly affected.

Validation of *LsPP2A*-*1*, *LsPP2AA3* and *LsTIP41* as good reference genes for diurnal studies was confirmed using a second target gene, the clock-controlled *FLAVIN*-*BINDING, KELCH REPEAT, F*-*BOX* (*FKF1*) [44]. Previous northern blotting analysis in *Arabidopsis* showed that *FKF1* expression levels in LDs start to increase at ZT 7 and reach a peak around ZT 10, and then decrease before dark at ZT 16 [45]. In our diurnal time-course the normalised expression of the lettuce homologue, *LsFKF1,* shows the same pattern verifying the suitability of the selected reference genes for such timecourse experiments.

## Conclusion

In this study reference genes suitable for use in diurnal and developmental timecourse experiments in lettuce have been identified. The references genes selected have been used to generate a much more detailed and robust analysis of *LsFT* expression levels in both diurnal and developmental timecourse experiments than has been achieved to date.

## Methods

### Plant materials, experimental conditions and tissue collection

Lettuce (*L. sativa*) seeds were sown in Levington F2 compost and covered with vermiculite, before being stratified in the dark at 4 °C for 48 h to achieve uniform germination. After stratification, trays were moved into long day (LD) conditions in the glasshouse (8 h dark and 16 h light) with supplementary lighting provided by high pressure sodium lamps when natural light levels went below 300 W/m^2^. Constant day/night temperature of 18 °C/15 °C was maintained. Plants were re-potted into 12.7 cm pots when they reached the 3–4 leaf stage.

For the diurnal experiment 24 plants were moved one week after the plants had started bolting from the glasshouse into controlled environment cabinets set for 16 h LD (daily light integral = 7.5 mol m^−2^ d^−1^) and a constant temperature of 18 °C. The lighting in the cabinets was provided by 60 W fluorescent tubes. Every 2 h one leaf from four different plants was collected and then pool together for a total of 15 samples. Sampling was performed in duplicate.

For the developmental timecourse experiment, the youngest newly expanded leaf from 5 different plants was harvested. The first harvest was carried out when the plants were 31 days old followed by a harvest each week for 12 weeks, the samples were always collected at the same time of day which was 14 h after lights on. Plants bolted at a timepoint between samples 6 and 7. Sampling was performed in duplicate.

### Total RNA isolation and cDNA synthesis

Total RNA was isolated from all samples collected using the Z6-extraction buffer method [35]. RNA concentration and quality was evaluated by spectrometry using A_260_/A_280_ ratios, RNA integrity was also evaluated running 1–2 µg of total RNA on 2 % agarose gel. Samples were DNase treated using TURBO DNA-freeTM. PCR amplifications were performed to prove the absence of DNA contamination in the RNA samples using primers for *L. sativa* elongation factor alpha (*LsELFα*) [lettuce EST database—CGP2 project:CLS_S3_Contig4947] (Additional file 1: Table S1). PCR reactions used KOD Hot Start DNA Polymerase and consisted of an initial denaturation at 94 °C for 2 min, denaturation at 94 °C for 15 s, annealing for 30 s, and extension at 72 °C for 30 s for 30 cycles. First-strand cDNA was synthesised from 3 µg cleaned total RNA using iScript™ cDNA synthesis kit following the manufacturer’s guidelines and subsequently treated with RNase H.

### Selection of candidate reference genes and primer design

A total of 10 candidate reference genes were chosen based on previously published papers using reference genes for developmental and diurnal time-course expression studies in other species (Table 1). The reference genes selected were; *PUTATIVE UBIQUITIN EXTENSION PROTEIN* (*UBQ1*), *UBIQUITIN*-*LIKE PROTEIN RUB2* (*UBQ7*), *ACTIN 2* (*ACT2*), *ACTIN PROTEIN CODING 12* (*ACT12*), *TUBULIN ALPHA*-*3* (*TUA*-*3*), *GLYCERALDEHYDE 3*-*PHOSPHATE DEHYDROGENASE*, *CYTOSOLIC* (*GAPDH*), *UBIQUITIN CONJUGATING ENZYME 9* (*UBC9*), *TIP41*-*LIKE PROTEIN* (*TIP*-*41*), *PROTEIN PHOSPHATASE 2A REGULATORY SUBUNIT A3* (*PP2AA3*) and *PROTEIN PHOSPHATASE 2A*-*1* (*PP2*-*A1*) [11, 36–42].

For each candidate reference gene, a blast search was carried out in the expressed sequence tag (EST) Compositae Genome Project Database (CGPDB 2013; http://cgpdb.ucdavis.edu/) against *L. sativa* coding DNA sequences using the *Arabidopsis* homolog as a query. The coding sequences of the matching contings were then retrieved and used to design primers using Primer3Plus (http://primer3plus.com/cgi-bin/dev/primer3plus.cgi) according to the following parameters: annealing temperature (Ta) of 60–67 °C with an ideal Ta of 65 °C, GC content of 45–55 %, an optimum primer length of 20–26 bp and a maximum product size of 200 bp. The secondary structure of the amplicons was examined using Mfold (http://mfold.rna.albany.edu/?q=mfold) so that amplicon sequences could be selected that didn’t have any secondary structure at the primer binding sites. Primer sequences and relevant information are shown in Additional file 1: Table S1.

The presence of any primer dimers and non-specific amplification was identified post-PCR through analysis of melting curve data and by sequencing the amplified products to confirm their identity.

### Real-time quantitative PCR

RT-qPCR analysis was conducted using the Bio-Rad CFX384 TouchTM Real-time PCR machine (Bio-Rad). Each reaction contained 5 μl Sso Advanced™ SYBRH Green Supermix, 0.5 μl cDNA, and forward and reverse primers in the appropriate concentration (Additional file 1: Table S1), in a total volume of 10 µl made up with sterile distilled water. Amplification conditions were as follows: 95 °C for 3 min followed by 40 cycles of 10 s at 95 °C and 30 s at the primer specific Ta (Additional file 1: Table S1). The melting curve was obtained by melting the amplified template from 65 to 95 °C increasing the temperature by 0.5 °C per cycle. No-template controls were included. Three technical replicates were used for each sample. Amplification efficiencies for all primer pairs were evaluated using serial tenfold dilutions of pooled cDNA. MiQE guidelines were followed for analysis [9].

### Reference gene stability

Each primer pair was screened for the best concentration to use in the PCR reaction. The geNorm algorithm [7] present in qBase Plus software version 2.5 (http://www.biogazelle.com/qbaseplus) was used to rank expression stability of the candidate reference genes across diurnal and developmental time-course samples. Using target- and run-specific amplification, geNorm generated an internal control gene stability measure (M) which represents the average pair-wise variation of each reference gene with all the other reference genes [7]. The M value for a good reference gene is arbitrarily set as below 1.5 [7]. The geNorm analysis, for the diurnal experiments, was initiated on 15 samples and 10 reference targets. The geNorm analysis, for the developmental experiments, was initiated on 12 samples and 10 reference targets. All samples were measured in the same run for a given reference target. MiQE guidelines were followed for analysis [9].

### Validation of reference gene stability

To test and validate the stability of the selected reference genes the expression profile of *L. sativaFT* (*LsFT*) [DNA Data Bank of Japan: AB602322] and *L. sativaFKF1* (*LsFKF1*) [43] were investigated in diurnal and developmental time-course experimental leaf tissue samples (Additional file 1: Table S1). RT-qPCR was performed as previously described with the appropriate primer concentration and annealing temperature (Additional file 1: Table S1). Normalised gene expression was determined using data from the three biological replicates by the geometric mean of the relative quantities for all and for the most stable reference genes according to geNorm results, and using target- and run-specific amplification with qBase Plus software version 2.5 (http://www.biogazelle.com/qbaseplus). MiQE guidelines were followed for analysis [9].

## Availability of data and materials

The datasets supporting the conclusions of this article are included within the article and its additional file.

## Additional files

10.1186/s13007-016-0121-y Primers used in this study.
10.1186/s13007-016-0121-y Candidate reference genes melting curve analysis. Melting curve analysis for each primer set for each candidate reference gene after 40 Real-time quantitative PCR cycles.
10.1186/s13007-016-0121-y Normalised expression levels of *LsFKF1*. *LsFKF1* expression levels normalised against the three more stable reference genes, *LsPP2A-1*, *LsPP2AA3* and *LsTIP41*, over the diurnal time-course. The white bar below indicates lights on, the black bar indicates darkness.
