# Supplementary material for: Selection of reference genes for diurnal and developmental time-course real-time PCR expression analyses in lettuce
Source: Plant Methods. 2016 Mar 22;12:21. doi: 10.1186/s13007-016-0121-y (PMC4804537; doi:10.1186/s13007-016-0121-y)
Supplement: Supplementary file 1 — 10.1186/s13007-016-0121-y Primers used in this study. [file 13007_2016_121_MOESM1_ESM.docx]

**Additional file 1 Table S1. Primers used in this study.**

| **Primers Name** | **Sequence (5’-­‐>3’)** | **Product Size**  **(bp)** | **Ta (°C)** | **Optimum primers Concentration (μMol)** |
| --- | --- | --- | --- | --- |
| **LsELFαF** | **CGGGTCAGATTGGAAACGGGTAT** | **326** | **61** | **0.5** |
| **LsELFαR** | **CTTCTTCGCAGCAGCCTTGGTGA** |  |  |  |
| **LsFT_RT_F** | **CTGGATGGCGTCAGAACTTCAATAC** | **122** | **61** | **0.4** |
| **LsFT_RT_R** | **TTATCTTCTTCGCCCACCAAACC** |  |  |  |
| **LsFKF1_RT_F** | **CAAACATCGAGCAACAACAACAAA** | **101** | **61** | **0.2** |
| **Ls FKF1_RT_R** | **CAACATCTCTGGGTGTCAATCGAG** |  |  |  |
| **LsUBQ1F** | **CCGAACTCTTGCTGACTATAACATC** | **122** | **65** | **0.4** |
| **LsUBQ1R** | **TCTTGTCTTGGTTGTATTTCCTAGC** |  |  |  |
| **LsUBQ7F** | **AGAACACTCGCTGATTACAACATCC** | **125** | **65** | **0.2** |
| **LsUBQ7R** | **GGCTCAATGTCGATTTCAATTTCTTT** |  |  |  |
| **LsACT2F** | **CAAGGGCAGTGTTTCCTAGTATTG** | **93** | **65** | **0.4** |
| **LsACT2R** | **CCTCATCTCCAACATAAGCATCTTTC** |  |  |  |
| **LsACT12F** | **ACCTCAGCAGAACGTGAAATTGTAA** | **120** | **65** | **0.2** |
| **LsACT12R** | **CTCGTAACTCTTCTCAACAGACGAA** |  |  |  |
| **LsTUA-3F** | **CTTCTTAGTGTTCAATGCTGTTGG** | **120** | **65** | **0.2** |
| **LsTUA-3R** | **GAAGGGTAGATAGTGAAACCGAGC** |  |  |  |
| **LsGAPDHF** | **TTAAAGGGTGGTGCTAAGAAGGTCAT** | **98** | **65** | **0.4** |
| **LsGAPDHR** | **AGCTCAGGTTTGTATTCGTTCTCATT** |  |  |  |
| **LsUBC9F** | **AAGGTATTGTTGTCGATTTGCTCTCT** | **118** | **65** | **0.4** |
| **LsUBC9R** | **TCCTTGCAGTTGTCTCATACTTGTTC** |  |  |  |
| **LsTIP41F** | **TTTGTATGGAGATGAATTGGCTGATA** | **90** | **65** | **0.2** |
| **LsTIP41R** | **CGTAAGAGAAGAAACCAACAGCTAGG** |  |  |  |
| **LsPP2AA3F** | **CATGCAATGGTTACAAGACAAGGTAT** | **80** | **65** | **0.2** |
| **LsPP2AA3R** | **CAAACTCCTCCGCAAGTCTCTTC** |  |  |  |
| **LsPP2A‐1F** | **ATTCATGGTCAATTCTACGATCTGGT** | **107** | **65** | **0.2** |
| **LsPP2A‐1R** | **GAATAATACCCGCGATCAACATAATC** |  |  |  |
