# Supplementary figures and images for: Selection of reference genes for diurnal and developmental time-course real-time PCR expression analyses in lettuce
Source: Plant Methods. 2016 Mar 22;12:21. doi: 10.1186/s13007-016-0121-y (PMC4804537; doi:10.1186/s13007-016-0121-y)

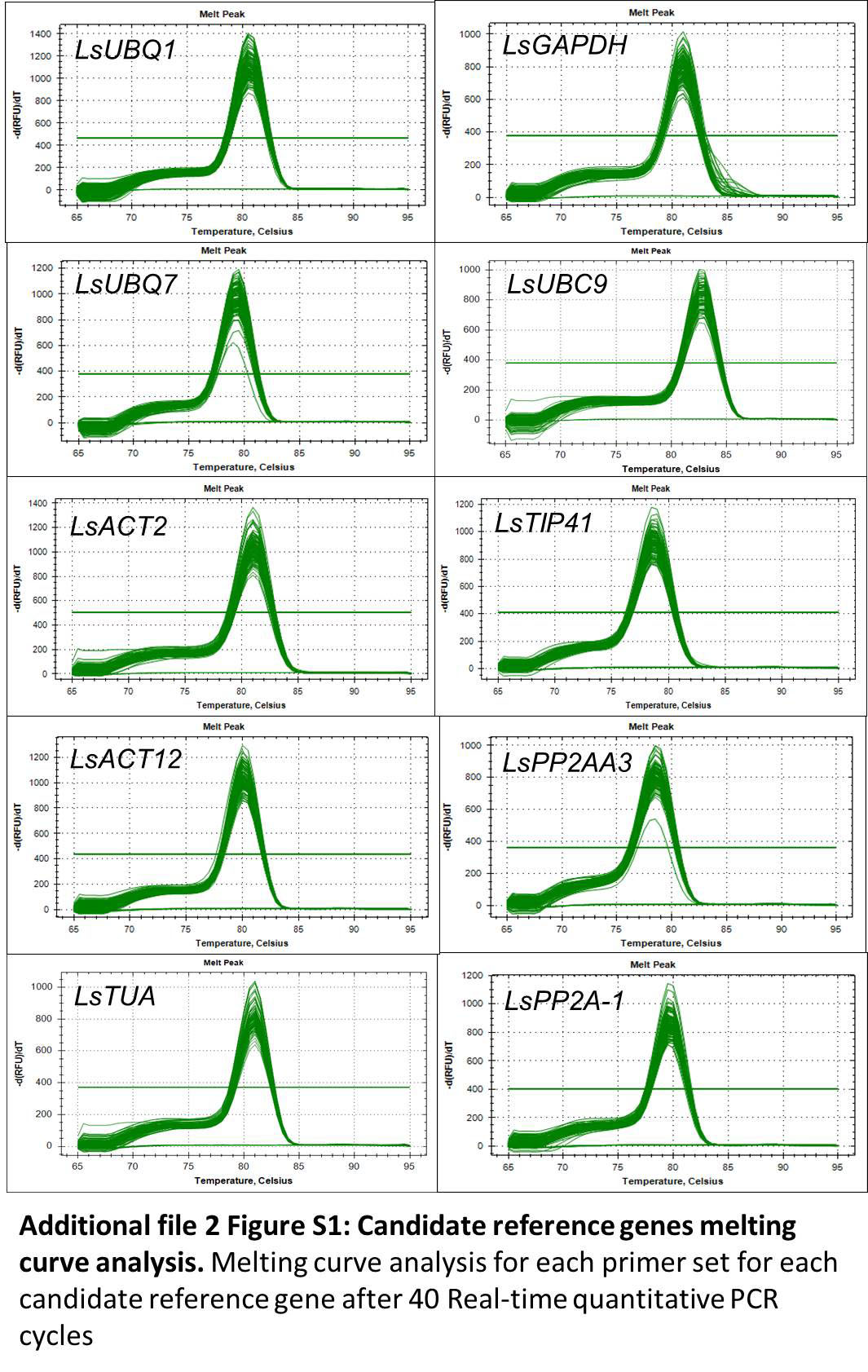

Supplement: Supplementary file 2 — 10.1186/s13007-016-0121-y Candidate reference genes melting curve analysis. Melting curve analysis for each primer set for each candidate reference gene after 40 Real-time quantitative PCR cycles. [file 13007_2016_121_MOESM2_ESM.jpg]

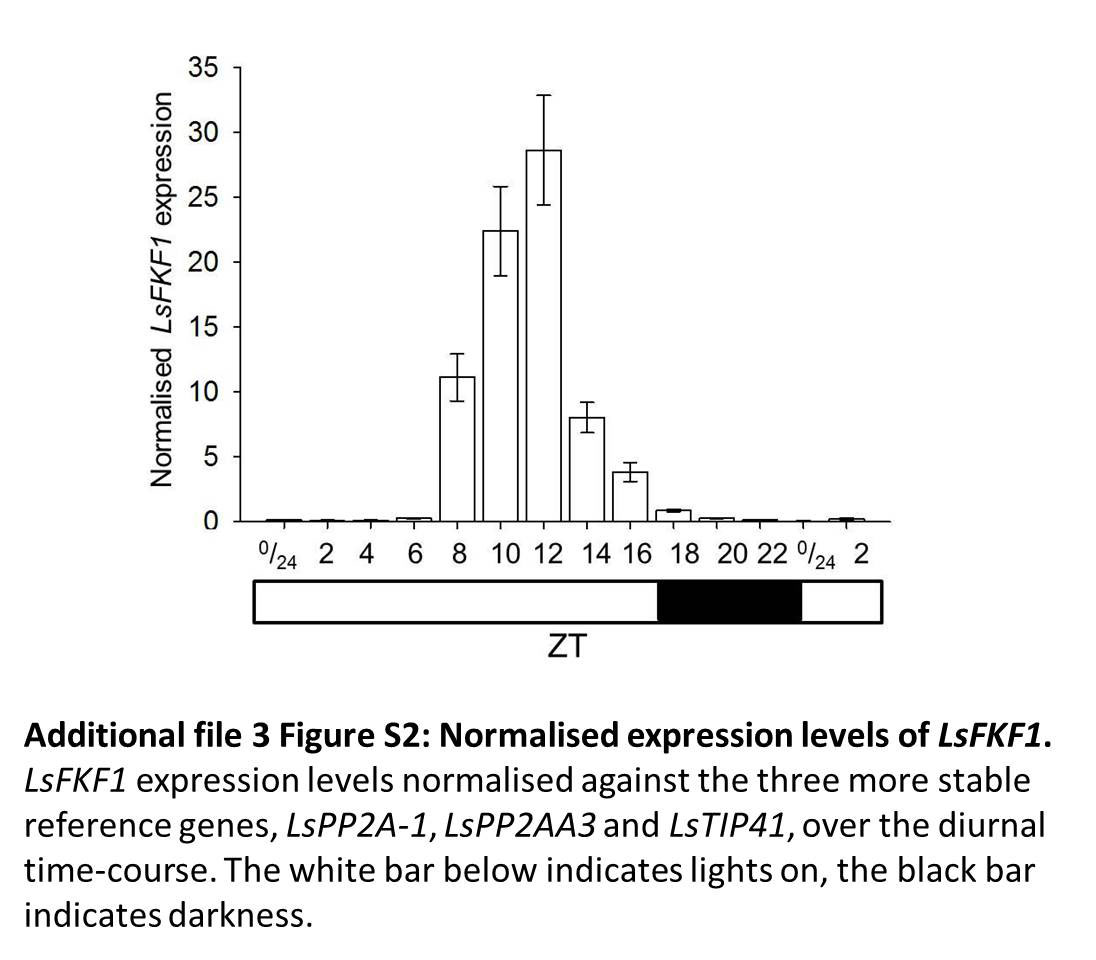

Supplement: Supplementary file 3 — 10.1186/s13007-016-0121-y Normalised expression levels of LsFKF1. LsFKF1 expression levels normalised against the three more stable reference genes, LsPP2A-1, LsPP2AA3 and LsTIP41, over the diurnal time-course. The white bar below indicates lights on, the black bar indicates darkness. [file 13007_2016_121_MOESM3_ESM.jpg]
